# mRNA Vaccine Designing Using Chikungunya Virus E Glycoprotein through Immunoinformatics-Guided Approaches

**DOI:** 10.3390/vaccines10091476

**Published:** 2022-09-06

**Authors:** Samavia Jaan, Aqal Zaman, Sarfraz Ahmed, Mohibullah Shah, Suvash Chandra Ojha

**Affiliations:** 1 Department of Biochemistry, Bahauddin Zakariya University, Multan 66000, Pakistan; 2 Department of Microbiology & Molecular Genetics, Bahauddin Zakariya University, Multan 66000, Pakistan; 3 Department of Basic Sciences, University of Veterinary and Animal Sciences Lahore, Narowal 51600, Pakistan; 4 Department of Infectious Diseases, The Affiliated Hospital of Southwest Medical University, Luzhou 646000, China

**Keywords:** chikungunya, mRNA vaccine, vaccinology, immunoinformatics

## Abstract

Chikungunya virus is an alphavirus transmitted by mosquitos that develops into chikungunya fever and joint pain in humans. This virus’ name originated from a Makonde term used to describe an illness that changes the joints and refers to the posture of afflicted patients who are affected by excruciating joint pain. There is currently no commercially available drug or vaccine for chikungunya virus infection and the treatment is performed by symptom reduction. Herein, we have developed a computationally constructed mRNA vaccine construct featuring envelope glycoprotein as the target molecule to aid in the treatment process. We have utilized the reverse vaccinology approach to determine epitopes that would generate adaptive immune reactions. The resulting T and B lymphocytes epitopes were screened by various immunoinformatic tools and a peptide vaccine construct was designed. It was validated by proceeding to docking and MD simulation studies. The following design was then back-translated in nucleotide sequence and codons were optimized according to the expression host system (*H. sapiens*). Various sequences, including 3′ and 5′ UTR regions, Kozak sequence, poly (A) tail, etc., were introduced into the sequence for the construction of the final mRNA vaccine construct. The secondary structure was generated for validation of the mRNA vaccine construct sequence. Additionally, in silico cloning was also performed to design a vector for proceeding towards in vitro experimentation. The proposed designed vaccine construct may proceed with experimental testing for further efficacy verification and the final development of a vaccine against chikungunya virus infection.

## 1. Introduction

An arbovirus designated as the chikungunya virus (CHIKV) is transmitted by the Aedes species of mosquito and is the cause of fever and disabling arthritogenic sickness affecting millions of people worldwide. It is a member of the Togaviridae family, genus Alphavirus, which includes O’Nyong-nyong, Mayaro, and the Ross River viruses, having a high capability of causing disease in humans [1]. It is a single-stranded RNA-containing virus that has a genetic material of roughly 12 kb in size. The 5′ORF, translation from the viral genome, encodes the non-structural elements nsP4, nsP3, nsP2, and nsP1, while 3′ORF, translation from the sub-genome, produces a polyprotein which is converted into various structural proteins, such as two peptides (6K and E3), envelope (E2 and E1), and capsid (C) [2]. E1 and E2 are viral spike proteins that enhance the virus’s ability to adhere to cell surface receptors and easily penetrate. The E1 envelope glycoprotein is a class II fusion protein that promotes membrane fusion driven by low pH during viral infection. The class I transmembrane glycoprotein, also known as E2 envelope protein, has been associated with receptor binding throughout the virus lifecycle [3]. The E1 protein is more significant as it uses a hydrophobic subunit to enter the target and refold itself into a hairpin-like structure.

The geographical spread of the CHIKV virus was aided by the E1-A226V mutation, which has high carrier competency for the *A. albopictus* mosquito. The E1-A226V, E2-I211T, and E1-T98A mutations highly increase the chances of CHIKV survival in *A. albopictus* [4]. The new Central African clade CHIKV (Cameroon strain) appears to be emerging dominantly with the mutations in recent studies. The majority of the chikungunya virus strains from Cameroon carried alterations in the envelope proteins (E1-A226V, E2-L210Q, and E2-I211T), which are known to raise CHIKV adaptability and infectious capacity in *Aedes albopictus* [5]. Additional data from similar alphaviruses such as Sindbis virus (SINV) and Semliki Forest Virus (SFV) suggest that at the 226th position of E1 glycoprotein, the valine residue regulates virus–cell membrane fusion’s reliance on cholesterol. As a result, the CHIKV E1-A226V mutation can change the biological features of E1 protein, which includes fusion ability, explaining the *A. albopictus* adaption process [6]. This makes the E1 an essential target protein for developing vaccines against chikungunya virus.

There is currently no available vaccine or proper drugs for the treatment of chikungunya viral infection. The only available vaccine in use contains an inactive entire virus, containing the complete chikungunya organisms that have had their genetic information deleted and hence cannot cause infections. People with weak immune systems and pregnant women are normally regarded as safe recipients of such immunizations. To avoid undesirable circumstances, various studies have suggested that in contrast to the prior one, the RNA vaccine is a more effective and productive therapeutic option. Thus, there is a dire need to bring to attention the development of an RNA vaccine against CHIKV infection [7]. Studies have shown that DNA vaccines result in the incorporation of the viral genome into the recipient genome, which could result in mutations. However, mRNA-based vaccines are designed to attack the cytoplasm and prevent the crossing of the nuclear membrane, which reduces the risk of nuclear genome integration [8]. The potential of the mRNA vaccine to induce both a humoral and a cell-mediated response with lower production costs and side effects and higher efficacy have certified it to win clinical examinations against a variety of deadly infectious illnesses [9]. Recent computational research has helped to design certain peptide vaccines, however there is currently no mRNA vaccine designed for CHIKV infection [10,11]. Herein, we employed computational techniques to examine the CHIKV enveloped glycoprotein and design an mRNA vaccine construct (CHIKV-mRNAV). We discovered the most antigenic and immunogenic B-cell as well as T-cell epitopes to form a peptide vaccine construct and back-translated it. Furthermore, we optimized codons in the conserved mRNA vaccine construct to improve the translation efficiency. We also used multiple bioinformatics techniques for predicting the peptide vaccine construct’s 3D structure together with docking studies and immunological simulation studies. The designed vaccine construct is proposed for further experimental efforts in producing an effective vaccine against CHIKV infection.

## 2. Materials and Methods

**Figure 1:**
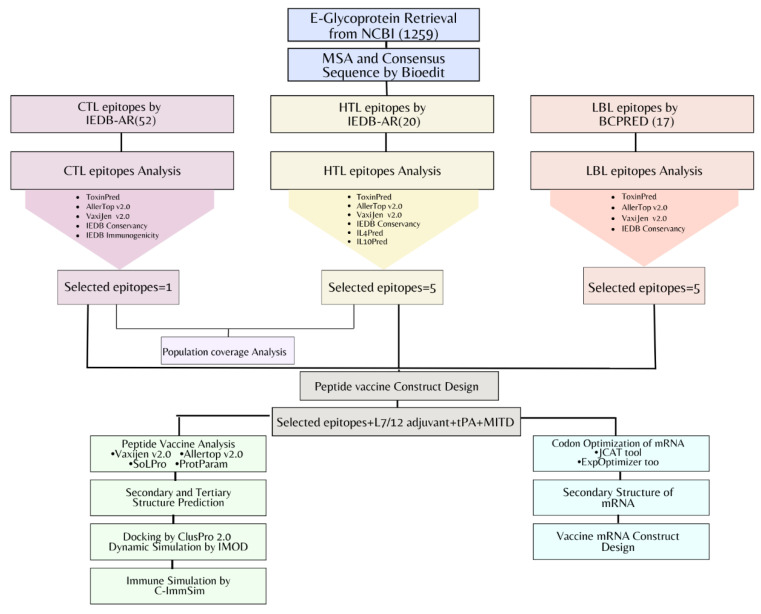
Flowchart of immunoinformatic and reverse vaccinology approaches followed for the potential mRNA vaccine design against CHIKV viral infection.

The pipeline of the mRNA vaccine construct designed against chikungunya virus has been outlined in Figure 1.

### 2.1. Retrieval of Envelope Glycoprotein Sequence

The complete amino acid sequences of the E1 glycoprotein chikungunya virus (CHIKV) were retrieved in the FASTA format from the National Centre for Biotechnology Information (NCBI).

### 2.2. MSA and Determination of Consensus Sequence

All the retrieved protein sequences were aligned by Multiple Sequence Alignment (MSA) using Bioedit software available with Clustal W [12]. After obtaining the aligned sequences, a consensus sequence was generated by the Bioedit built-in option for creating a consensus sequence. The non-conserved amino acids were manually replaced by “X” for the prediction of only highly conserved epitopes in further steps.

### 2.3. T-Lymphocytes Epitopes Prediction

The CTLs (cytotoxic T-cells) and HTLs (helper T-cells) epitopes can associate with MHC molecules and trigger a cell-mediated immune response. The epitopes of CTLs were predicted using the NetMHCpan 4.1 method from the IEDB-Analysis Resource [13]. The MHC-I allele set of coverage > 97% and peptide length “9-mer” were selected as per a previous study [12]. The resulting epitopes with “X” amino acids were excluded and the remaining were screened based on the rank < 0.2, as they were considered to have the highest binding affinity with MHC-I molecules. Similarly, the epitopes of HTLs were also predicted using the IEDB recommended 2.22 methodology, available in the IEDB-AR database. A reference MHC-II allele set of >99% population coverage and a length of predicted epitopes of “15-mer” were selected. The resulting epitopes with “X” amino acids were deleted and the remaining were filtered based on the rank < 2.0 in the case of MHC-II binding epitopes. The lower the rank, the higher the ability of the predicted epitope to interact with MHC molecules [14,15,16].

### 2.4. T-Lymphocytes Predicted Epitopes Analysis

For the selection of the final promising T-cell epitopes, various parameters were checked. AllerTop v2.0 [17] and Vaxijen v2.0 [18] servers were used to determine the allergenicity and antigenicity of the predicted epitopes, respectively. For toxic reaction prediction, the Toxinpred server was utilized. The conservancy analysis was carried out to select the epitopes with >90% conserved sequences. Some additional parameters were also determined for the analysis of different T-cell epitopes. In the case of MHC-I-interacting T-cell epitopes, the Class-I immunogenicity server provided by the IEDB database was utilized. Similarly, MHC-II-interacting T-cell epitopes were additionally checked for IL4 and IL10 induction abilities by the respective IL4Pred and IL10Pred servers. Final selected T-cell epitopes were proceeded for designing the vaccine construct against pathogen infection.

### 2.5. Population Coverage Analysis (PCA) of T-Lymphocytes

The population coverage tool of IEDB has been employed to determine the population percentages for predicted epitopes in selected MHC-I- and II-interacting T-cell epitopes with their corresponding majorly occurring MHC molecules. The population coverage analysis for the world’s greater infection rate countries, including South Asia, i.e., India, and Pakistan was explored [19].

### 2.6. B-Lymphocytes Epitopes Prediction and Analysis

The linear B-lymphocytes (LBL) epitopes represent parts of pathogen protein sequences that can bind to the B-cells and cause an antibody response. These were determined using an online available BCPREDS server for B-cell epitopes prediction [20]. A threshold of 75% specificity was selected for the preference of high-affinity epitopes. The antigenicity, allergenicity, and toxicity of B-cell epitopes were estimated using Toxinpred, AllerTop v2.0, and Vaxijen v2.0 servers [21]. Moreover, the conservancy analysis was also conducted to obtain >90% conserved epitopes from the protein sequence [22].

### 2.7. Multi-Epitope Vaccine Construct Design

The final selected epitopes of T- and B-cells were combined using linkers in designing a vaccine construct. HTL epitopes were joined using “GPGPG linker”, whereas CTL epitopes were fused by “AAY linker”. The remaining B-cell epitopes were combined by the usage of “KK linker”. Adjuvant and MITD sequences were also added to boost the immune reaction produced by the vaccine construct. The ribosomal L7/L12 protein sequence was used as an adjuvant in the respective vaccine construct that was linked using the “EAAAK linker”. Moreover, a signal tPA peptide (tissue plasminogen activator) was also introduced at the N-terminal end to aid in the transport of the vaccines out of the cell according to the required conditions [23].

### 2.8. Peptide Vaccine Construct Analysis

The peptide vaccine construct was evaluated using various parameters before proceeding to design the mRNA vaccine construct. The Expasy Protparam server was utilized to calculate the protein vaccine constructs’ molecular weight, the total number of amino acids, the total number of positive and negative charged amino acids, the aliphatic index, the theoretical isoelectric point, and the GRAVY score [24]. The Vaxijen v2.0 server with a threshold of >0.4 was used to calculate the antigenicity of the vaccine construct. The allergenicity was assessed using the AllerTop v2.0 and the SOLpro server was preferred for the prediction of solubility during heterologous expression inside the host. The acceptable solubility overexpression threshold was kept as >0.5 [25].

### 2.9. Prediction of Secondary and Tertiary Structures of the Vaccine Construct

The secondary structure of the peptide vaccine was generated via the SOPMA server. By employing the given server for computing, regions forming helix, extended strand, and coil structures were predicted [26]. The tertiary structure was predicted using the TrRosetta server [27], which was subsequently refined by the Galaxyrefine webserver [28]. Furthermore, the resulting structure was validated for 3D quality using a Ramachandran plot by PROCHECK at the SAVES server and quality factor at the ERRAT Web server [16].

### 2.10. Conformational B-Cell Epitopes Prediction

The conformational epitopes were also predicted as they help to determine surface-exposed and solvent-accessible epitopes of B-cells. In the vaccine construct, conformational B-cell epitopes were predicted by using the Ellipro server available at IEDB-AR [29].

### 2.11. Molecular Docking and Dynamic Simulation Studies

The vaccine construct was docked with MHC molecules, i.e., TLR2 and TLR4, to test the vaccine’s affinity with humoral immune response cells. The protein data bank (PDB) was used to download the crystal structures of TLR2 and TLR4 used in the analysis. The protein structures were prepared before processing for docking using the MOE software package. The co-crystallized ligands and water molecules were removed followed by protonation through built-in MOE options. Moreover, the energy minimization algorithm tool was used to minimize the energy of protein structures. MOE calculates protein energy (in kcal/mol) using the MMFF94x force field and the conjugant gradient method [30].

The prepared vaccine construct then proceeded for docking studies via ClusPro 2.0. The ClusPro 2.0 proceeds through three computational steps, including: (I) rigid body docking by using the fast Fourier transform (FFT) correlation method, (II) RMSD-based clustering of the configurations generated to find the biggest cluster that will reflect the complex’s likely models, and (III) fine-tuning of selected structures [31]. Herein, we have employed ClusPro 2.0 for the molecular docking study of a peptide vaccine construct with TLR4 and TLR2 receptors. The resulting MD complexes were analyzed afterward using DIMPLOT, available in LIGPLOT software [32]. Furthermore, root-mean-square, deformability, covariance, and B-factor fluctuation analyses were performed on the MD complexes to identify any residues that remained deformed or unstable after the coarse-grained MDs. The iMODS software was used for these particular analyses [33].

### 2.12. Immune Simulation Studies

The C-ImmSim server [34] was chosen to execute online dynamic immune simulation reports to better validate the peptide vaccine construct’s immunological response. A total of 3 doses (01, 84, and 168) with 1000 vaccine units and no LPS were given in a duration of 4 weeks, having a volume = 10, random seeds = 12,345, and number of steps = 1050. The remaining parameters were kept at the default [35].

### 2.13. Back-Translation and Codon Optimization of Vaccine Construct

The peptide vaccine construct was back-translated according to the expression host (*H. sapiens*) using JCAT and ExpOptimizer tools. The effective translation ability of mRNA was determined by the CAI (codon adaptation index) and GC content percentage obtained in the results. The best optimized mRNA sequence was selected and assessed using the Rare Codon Analysis Tool available at the GenScript site. Herein, the presence of unusual codons was represented by the Codon Frequency Distribution (CFD) factor [23].

### 2.14. Secondary Structure Prediction of mRNA Sequence

The secondary structure of messenger RNA is critical in the biosynthesis of proteins. Its negative influence on translation can significantly decrease the protein yield by halting or obstructing ribosome initiation and action along the mRNA, making it a significant factor in gene regulation. Several algorithms can predict secondary structure formation by calculating the minimum free energy of RNA sequences. The secondary structure of the mRNA sequence was predicted using RNAfold and mfold webservers [36,37].

### 2.15. mRNA Vaccine Construct Design

The mRNA vaccine construct sequence was designed as follows: **5′** m7GCap-5′ UTR-Kozak sequence (containing start codon)–optimized mRNA (tPA (signal peptide)–EAAAK linker–L7/L12 adjuvant)–GPGPG linker–HTL epitopes–KK linker–LBL epitopes–AAY linker–CTL epitopes–AAY linker–MITD sequence)–stop codon–(3′UTR)_2_–poly (A) tail 3′. The conventional mRNA vaccine construct was developed that comprised 5′ cap to prevent degradation and aid in translation. The 5′ and 3′ UTR regions were added from beta-globulin protein to increase translation efficacy. The Kozak sequence was also introduced that contained a start codon. The optimized mRNA sequence was added, and poly (A) tail was lastly added at the 3′ end to increase the RNA stability [38].

### 2.16. In Silico Plasmid Design for Cloning

The mRNA sequence was optimized according to *E. coli K12* for cloning using the JCAT tool. Using the SnapGene tool, a recombinant plasmid with mRNA vaccine construct (CHIKV-mRNAV) was designed. The NdeI restriction sites at the N terminal and XhoI restriction sites at the C terminal were introduced into the pET28a (+) vector with a circular plasmid setting. The plasmid DNA and vaccine construct were ligated using the “one fragment ligation” option. The resulting ligated fragment has been highlighted in red.

## 3. Results

### 3.1. Determination of Consensus Sequence

The available 1259 CHIKV envelope glycoprotein sequences from the NCBI database were subjected to multiple sequence alignment using Clustal W. Most of the amino acids in MSA aligned sequences were found to be conserved. A consensus sequence was generated using the MSA aligned file by Bioedit software, where all non-conserved amino acids were manually replaced by “X” for recognition of variable regions.

### 3.2. T-Lymphocytes Epitopes Determination

As the epitope for vaccine construct should be conserved in all the strains of the virus, thus only the E glycoprotein consensus sequence was utilized for epitopes’ identification. The T-cell epitopes were elucidated by using NetMHCpan 4.1 methodology that was accessed from the IEDB database. Using a comprehensive HLA allele reference set, it identified 52 distinct CTL epitopes with a predictive rank ≤ 0.2 (Supplementary Table S1). The IEDB class I immunogenicity tool detected 22 epitopes as positive regarding immunogenicity. Following that, the epitopes of T-cells were submitted to the population conservancy analysis, with only 2 out of the 22 epitopes exhibiting more than 90% conservancy via the IEBD conservancy prediction tool. These epitopes were also forwarded to VaxiJen v.2.0 (0.4 threshold) screening for their antigenicity estimation. To check their allergenic and toxic potential, the epitopes were additionally examined for toxicity and allergenicity utilizing the Toxinpred and AllerTOP v.2.0 servers. Out of these, one epitope was found to be the best final epitope for peptide vaccine construction following the mentioned parameters (Table 1).

For class II MHC epitopes’ prediction, we employed the MHC II binding predicting tool in IEDB. A total of 20 unique epitopes with an adjusted rank ≤ 2.0 were chosen from the prediction results, as the lesser percentile rank implies good interaction affinity (Supplementary Table S2). They were screened for conservancy using the IEDB conservancy prediction tool, resulting in 12 epitopes with more than 90% conservancy. These T-helper cell epitopes were further tested for antigenicity using the VaxiJen v2.0 server, as well as allergenicity and toxicity using the AllerTOP v2.0 preceded by the ToxinPred server. The IL10- and IL4-inducing capability analyses were also conducted employing the IL10pred and IL4pred servers, respectively. As a result, five epitopes were chosen for further analysis based on their non-allergenicity, non-toxicity, antigenicity, and ability of at least one interleukin-type induction (Table 2).

### 3.3. Population Coverage Analysis (PCA) of Selected T-Lymphocyte Epitopes

A variety of possible HLA alleles exist in various cultures, with varying frequencies. To manufacture a vaccine without any of the ethnically biased population coverage, the projected affinity ability of epitopes to maximal MHC molecules could be employed to predict the population coverage in a variety of ethnicities having an infection possibility. The IEDB population coverage investigation focused on South Asia’s population coverage of the 5 HTL and 1 CTL epitopes that were included in the vaccine construct (Supplementary Table S3).

### 3.4. B-Lymphocytes Epitopes Determination

The linear B-cell epitopes were predicted via the BCPRED server. Based on the findings, 17 potential B-cell epitopes were chosen for further investigation. Out of all selected epitopes, five epitopes were shortlisted based on their conservancy, non-allergenicity, antigenicity, and non-toxicity profiles (Table 3).

### 3.5. Peptide Vaccine Construction and Analysis

**Figure 2:**
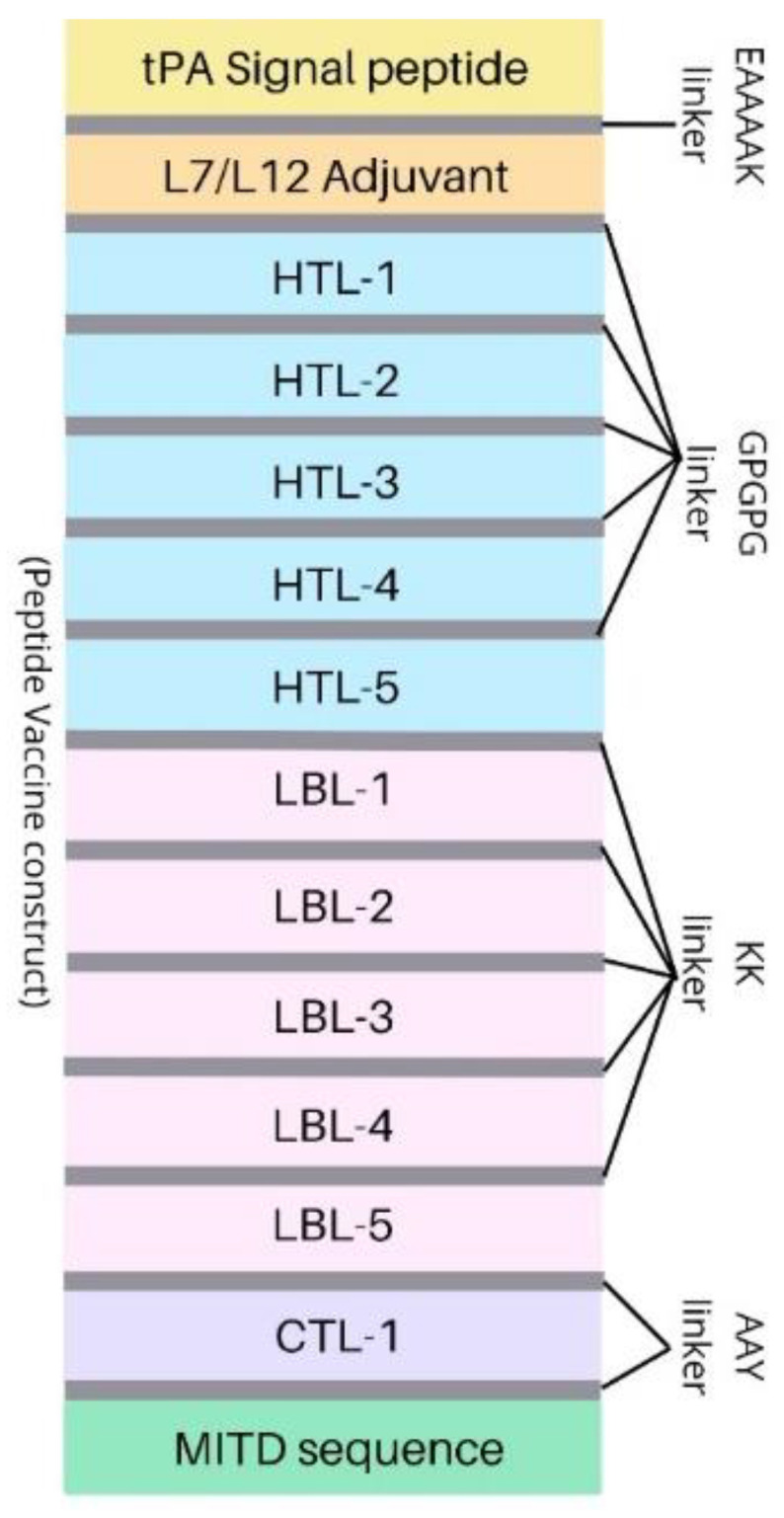
Peptide vaccine construct design for potential mRNA vaccine.

The vaccine design was performed using 5 linear B-cell epitopes, 5 HTL epitopes, and 1 CTL epitope, respectively. Adjuvants, linkers, and the tPA signal peptide were also introduced into the vaccine construct. The MITD sequence was also incorporated into the vaccine to boost the immune response. The construct consists of: signal peptide-linker (EAAAK)–50S ribosomal protein L7/L12 adjuvant-linker (GPGPG)–HTLs-linker (KK)–LBLs-linker (AAY)–CTLs-linker (AAY)–MITD sequence (Figure 2).

The Protparam server was used to evaluate the chemical and physical features of the peptide vaccine construct created from postulated epitopes. Protparam estimates properties such as the theoretical isoelectric point, relative molecular weights, aliphatic index, grand average of hydropathicity (GRAVY), and many others, including the number of positively and negatively charged amino acids. Additionally, other factors, e.g., antigenicity by Vaxijen v2.0, allergenicity by AllerTop v2.0, and solubility upon overexpression by SolPro servers, were also calculated (Table 4).

### 3.6. Peptide Vaccine Structure Prediction and Validation

**Figure 3:**
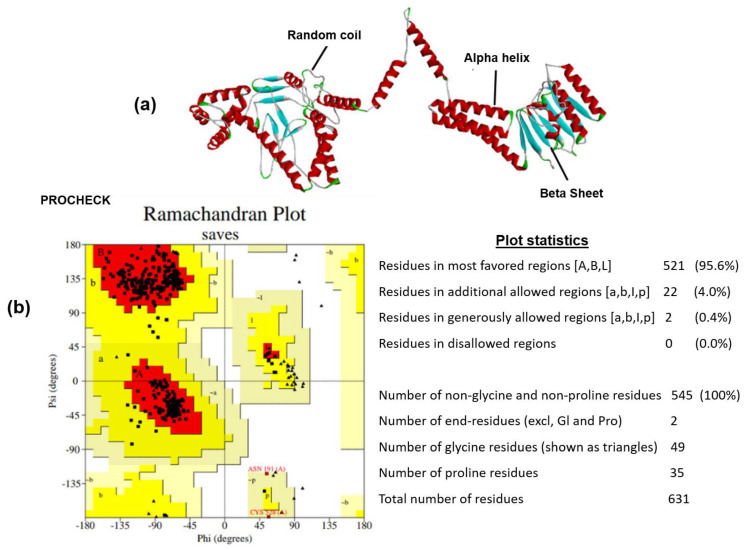
Validation results for the designed tertiary structure of the peptide vaccine construct. (**a**) Three-dimensional structure with highlighted alpha helix, beta sheet, and coil structures. (**b**) Ramachandran plot indicating 95.6% residues in most favored region.

The secondary structure of the designed vaccine construct was identified using the SOPMA server. The predicted structure was shown to have a 39.62% alpha helix, a 19.65% extended strand, a 11.41% beta turn, and a 29.32% random coil structure (Supplementary Figure S1). The model of vaccine constructs was generated using the Trrosetta server, which was further subjected to refinement in the GalaxyRefine webserver. Tertiary structure verification is a significant stage of the model designing method because it identifies potential errors in predicted 3D models. Thus, the predicted three-dimensional structure was validated by the ERRAT server, where the quality factor obtained was 95.86, indicating good quality of the 3D structure. Another validation parameter, the Ramachandran plot, was also employed to cross-check the refined 3D structure. The Ramachandran plot describes the model’s quality by displaying the percentage of residues in favorable, allowed, and disallowed regions. The refined 3D structure was exposed to the Ramachandran plot analysis using the SAVES webserver, where the results showed 95.6%, 4.0%, and 0.0% of residues in favored, allowed, and disallowed regions, respectively. The results indicate overall good quality of the predicted 3D structure (Figure 3).

### 3.7. Conformational B-Cell Epitopes Prediction

The conformational B-cell epitopes were predicted in the designed vaccine construct by the Ellipro server. Conformational epitopes are spatially close residues of exposed antigens that form separate segments of solvent-accessible regions when viewed in sequences. This accessibility helps in Ag–Ab complex formation when a vaccine is injected, and thus it is important to be checked in the vaccine construct. A total of 9 conformational B-cell epitopes were identified in the vaccine construct, indicating the vaccine’s ability to effectively stimulate the B-cell response (Supplementary Table S4). The highest-scoring conformational B-cell epitopes have been depicted in Supplementary Figure S2.

### 3.8. Docking and MD Simulation

**Figure 4:**
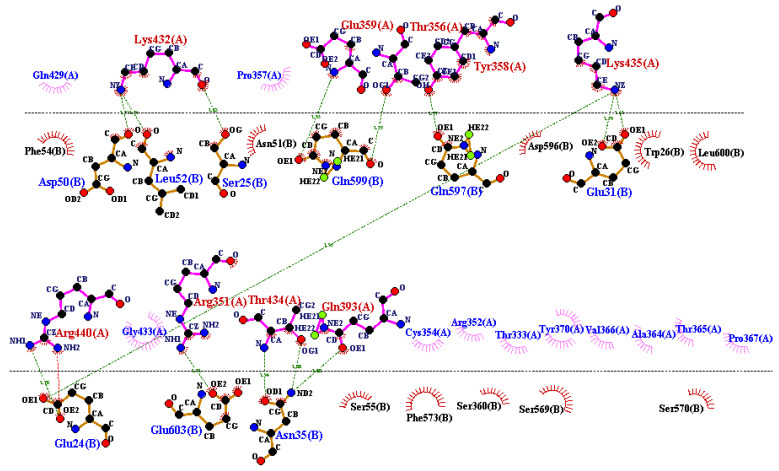
Possible interactions between vaccine construct and TLR4 obtained by docking studies and visualized through the DIMPLOT server. The green lines show hydrogen bond interactions between TLR4 receptor’s B chain amino acids (labeled with A at the top section of the lines) and the vaccine construct amino acids (labeled with B at the bottom section of the lines).

The vaccine construct was subjected to docking with TLR2 and TLR4 receptors to check the possible interactions between vaccine and HLA molecules. The receptor proteins from PDB cannot be used directly for molecular docking since they might be linked with complex compounds, co-crystallized small molecules, molecules of water, ions, and co-factors, necessitating the preprocessing by adding H+ ions, clearing nearby water molecules, adding polar charges, and separating co-bound ligand(s). Following the preprocessing, they were saved in pdb format. We subjected the vaccine construct to the molecular docking analysis with both TLR2 and TLR4 in the ClusPro 2.0 server. The resulting MD complexes were examined for potential interactions across the structures. When there are more hydrogen bonds plus pi–pi interactions, salt bridges, and hydrophobic interactions, a complex is considered more stable. The vaccine in the molecular docking showed the lowest energy score of −1041.1 with TLR4 and −999.7 with TLR2, respectively. The interaction analysis showed the good binding affinity of the complexes, especially TLR4 with lower energy scores; thus, it was proceeded for MD simulation by the IMODS server. The best complex interaction was visualized by DIMPLOT provided in the LIGPLOT software package (Figure 4).

**Figure 5:**
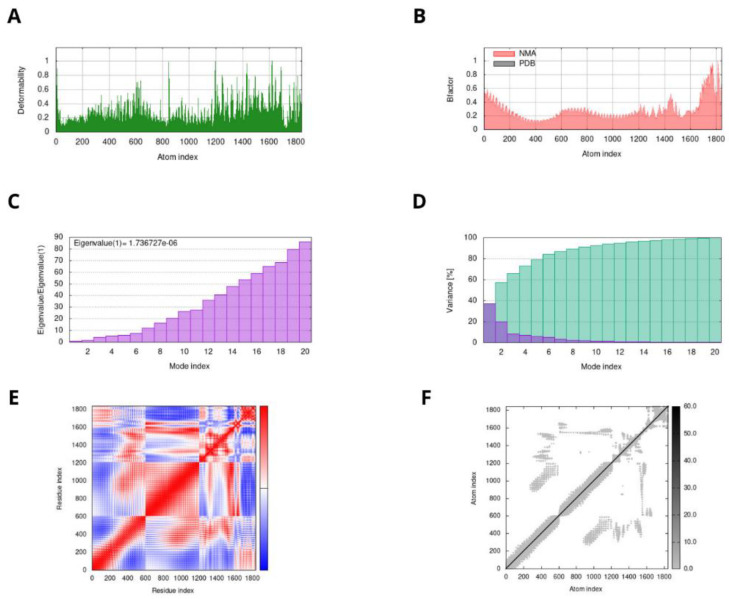
MD simulation results of peptide vaccine construct–TLR4 complex. The stability of the protein–protein complex was examined by: (**A**) deformability, (**B**) B-factor values, (**C**) eigenvalue, (**D**) variance, (**E**) covariance of residue index, and (**F**) elastic network analysis.

The results of normal mode analysis (NMA) and molecular dynamics simulation of the vaccine construct and the TLR-4 docked complex were obtained by the IMODS server. Deformability is a way of measuring a molecule’s ability to modify at any of its residues. This is most noticeable in the form of “highest peaks”, associated with high deformability areas in the represented graph. The second factor was the B-factor that was estimated by determining the PDB structure and running it through normal mode analysis (NMA), after which multiplying the NMA mobility by 8pi2. Other parameters include the eigenvalue of the complex, that was obtained as “1.73672710^−06^”, and the covariance matrix that showed how the complex’s residues were associated. The higher the correlation, the stronger was the complex. The red, white, and blue colors show a valuable correlation, no correlation, and anticorrelations between the residues, respectively (Figure 5).

### 3.9. Immune Simulation

**Figure 6:**
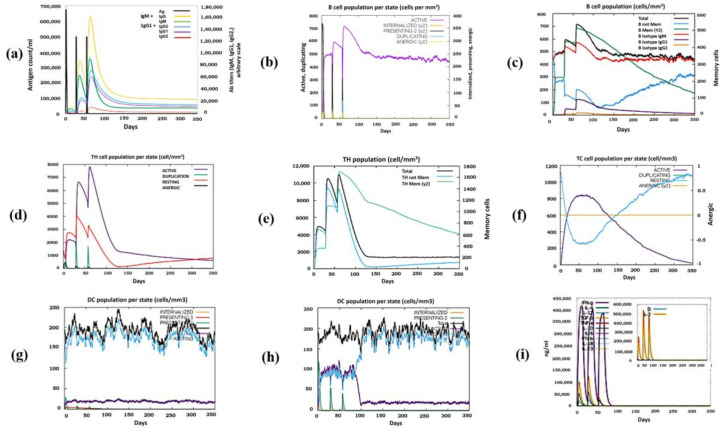
Immune simulation of peptide vaccine construct in three injections by C-immsim server. (**a**) Primary Antibody responses. (**b**) Active B-cells profile (**c**) Memory B-cells profile. (**d**) Active Helper T-cells profile. (**e**) Memory Helper T-cells profile. (**f**) Active Cytotoxic T-cells profile. (**g**) Dendritic cells profile. (**h**) Macrophages response profile. (**i**) Interleukins and cytokines profile.

The immune simulation profile was checked by using the C-ImmSim webserver. The graph “a” in Figure 6 represents the primary antibody response of IgM and IgG, where the rate of IgM + IgG increased significantly following the third vaccine injection. The graph “b” represents the B-cell immune response with indication of an active B-cell response that remains constantly high after the third injection. Similar to graph “b”, graph “c” presents the complete B-cell profile changes by introduction of the vaccine after eleven weeks. The graphs “d” and “e” show a high increase in the production of helper T-cells, while graph “f” presents the cytotoxic T-cell duplication profile, all indicating positive immune results. The graphs of ”g” and “h” represent profiles of dendritic cells and macrophages throughout the injection phases of the vaccine in the body. The last graph “i” represents the interleukin levels and danger levels “D”, where the peak for danger is too low, indicating a positive response for vaccine usage (Figure 6).

### 3.10. Optimized mRNA Determination

To perform the codon optimization, two different tools were used: the Java Codon Adaptation Tool (JCat) and the ExpOptimizer tool. The results of these tools were further evaluated by resulting parameters, such as the codon adaptation index (CAI) and GC content (percentage values). The optimized mRNA sequence obtained from the JCAT tool showed a high CAI score, and the GC content was also found to be within the appropriate range. This optimized mRNA was further validated using the Rare Codon Analysis Tool by the GenScript server. There, the value of an unusual codon was observed by factor CFD, that came to be 0.00%. This indicated the overall high translation efficacy of the optimized mRNA sequence (Supplementary Table S5).

### 3.11. Secondary Structure Prediction of Optimized mRNA

The secondary structure was predicted using the free minimum energy approach, and it is preferred to verify the structure using more than one server. Herein, the secondary structure prediction was performed employing two webservers, i.e., mfold and RNAfold servers. The resulting free energy obtained by folding the mRNA sequence in mfold was −796.90 kcal/mol. The RNAfold afforded a free energy value of −789.60 kcal/mol, indicating approximately the same value as the initial mfold value, thus predicting a good-quality secondary structure.

### 3.12. mRNA Vaccine Construct Design

The final mRNA vaccine construct sequence was designed as follows: 5′ m7GCap-5′ UTR–Kozak sequence (containing start codon)–optimized mRNA (tPA (signal peptide)–EAAAK linker–L7/L12 (adjuvant)–GPGPG–SGRPIFDNKGRVVAI–GPGPG–ERMCMKIENDCIFEV–GPGPG–ITCEYKTVIPSPYVK–GPGPG–VYKGDVYNMDYPPFG–GPGPG–YACLVGDKVMKPAHV–KK–ASLQHTAPFGCQIATNPVRA–KK–VGPMSSAW–PFDNKIVVYKG–KFTHEKPEGYYNWHHGAVQY–KK–ERIR–EATDGTLKIQVSLQI–KK–MCARRRCITPYELTPGATVP–AAY–KYDLECAQI–AAY–MITD sequence)–stop codon–(3′UTR)2–poly (A) tail (120 bases) 3′. In the construct, the 5′ Cap will mediate translation factor binding by preventing mRNA degradation, the 3′ and 5′ beta-globin UTR will improve translation efficacy, the Kozak sequence will function as the initiation site of the protein translation in many eukaryotic mRNA transcripts, and the tPA signal sequence will guide the target protein into the cellular secretion pathway. To improve mRNA stability and the translation rate, a poly (A) tail of 120 bases was added at the 3′ end (Supplementary Figure S3).

### 3.13. rPlasmid Design for In Vitro Cloning

The mRNA vaccine sequence was optimized according to the *E. coli K12* strain using the JCAT server. The sequence was then evaluated by checking the two parameters, i.e., the CAI index and the GC content. It was found that the optimized sequence had a CAI index of 0.95 and a GC content of 49.71%, indicating a stable optimized mRNA sequence. This mRNA sequence was cloned in the pET 28a(+) vector using restriction sites of NdeI (CATATG) and XhoI (CTCGAG) endonucleases, with an approximate size of 1893 bps. The in silico cloning was performed utilizing SnapGene software. As a result, a ligated rPlasmid was obtained that showed the possibility of in vitro cloning of the mRNA vaccine construct for commercial purposes (Supplementary Figure S4).

## 4. Discussion

Chikungunya virus is considered one of the most important arboviruses in terms of public health. It belongs to the Togaviridae family and the alphavirus genus, and it causes chikungunya fever (CHIKF), an arthritogenic disease. It is distinguished from other arbovirus infections by a severe and painful arthralgia that could last for months or years in some individuals. Despite the significant social and economic costs of CHIKV infection, no vaccine or drug treatments are presently available. The immunization against CHIKV infection is only dependent on an inactive virus vaccine that can be injected in limited types of individuals [39]. In this situation, secure and reliable CHIKV immunity development could contribute significantly to the virus’s complete annihilation. Since the first successful case of mRNA therapeutics in 1990, mRNA vaccines against influenza, rabies, HIV-1, Zika, and other viruses have symbolized a useful and incredibly effective subset of vaccines [40]. The mRNA vaccine has the potency to be more widely accepted than the currently accessible inactive virus vaccine. mRNA are translated into target proteins once they are introduced into the host cells. The proteins are then processed within the cell’s environment. To trigger a significant T-cell-mediated immunological response, the synthesized antigenic peptides are delivered to the surface of the cell via MHC molecules. By activating the B-cells to create the neutralizing antibodies, helper T-cells speed up the clearing of the circulating infections. Furthermore, phagocytes are activated by inflammatory cytokines generated by helper T-cells, such as interferon gamma (IFN-c) [41].

This study used the CHIKV envelope glycoprotein for designing an mRNA vaccine against CHIKV infections. At first, in silico tools were used to make the conserved E glycoprotein sequence that was proceeded for immunoinformatic analysis to construct a vaccine sequence. The T- and B-cell epitopes were determined using the IEDB-AR and various other parameters, including the antigenicity, allergenicity, conservancy, and toxicity analysis. Some additional parameters were also checked that were associated with each category of epitopes. After a careful analysis, the final promiscuous B- and T-cell epitopes were linked using linkers to design a vaccine construct. Other sequences such as the L7/L12 ribosomal adjuvant and MITD sequence were also added to boost the immunogenic response by the vaccine construct. Another amino acid chain was the signal peptide derived from *H. Sapiens* tissue plasminogen activator (tPA). The tPA signal sequence improves the vaccine’s immunogenicity. The designed vaccine construct was checked for various parameters to ensure its immunogenic capabilities by using Protparam, Vaxijen, Allertop, and Solpro servers. The secondary and tertiary structures were also predicted and validated by different servers. The refined 3D structure of the vaccine construct was docked with majorly occurring immune receptors in humans, i.e., TLR2 and TLR4, by the Cluspro v2.0 server. Results showed that the vaccine construct had a higher affinity with TLR4, and thus the vaccine construct–TLR4 complex was forwarded to MD simulation studies. Herein, the simulation graphs were generated by the iMODs server that included deformability, B-factor, and other analysis data, indicating positive results for the vaccine construct–TLR4 complex. After all these confirmations, the vaccine construct was finally checked for an immune response by the C-ImmSim server, which creates an immune profile based on injections administered at various intervals. The immune profile showed increased T- and B-cell responses, which led to the use of this vaccine construct in designing a mRNA vaccine sequence.

The sequence of the mRNA vaccine was made using various sequences, such as the 5′ and 3′ UTR regions, 5′ cap, the back-translated vaccine construct, the poly (A) tail, etc. The 5′ and 3′ UTR regions hold great importance in the design of the vaccine. In our study, we utilized the 5′ and 3′ UTR regions of the beta-globin gene in the vaccine construct. These regions are important for improving the translation efficiency of the mRNA. The size of the poly A tail also affects the translation efficiency. The results of the study revealed that the mRNA with this tail of 120 bases had prolonged protein expression. For the 5′ end-capping, we chose to use cap 1 (7-methyl-GpppN20-O-methyl). This allowed us to increase the half-life of mRNA by synergizing the 3′ end poly A tail and the 5′ cap. The Kozak sequence was also used to improve the translation of the vaccine [14]. The final constructed mRNA sequence was cloned in *E. coli K12* strain using the SnapGene tool for commercial production purposes. The results showed a stable plasmid containing the designed mRNA vaccine sequence, which was successfully ligated in it. It may be possible for the constructed vaccine sequence to be cloned and expressed in the *E. coli K12* strain for increased production of the vaccine. This study aimed to help in the fast and accurate production of a suitable CHIKV vaccine that may further aid in a future vaccine development process. Further in vitro and in vivo studies are needed to investigate the use of the constructed mRNA sequence at the commercial level.

## 5. Conclusions

The primary benefits of using mRNA-based viral infection vaccines are their stable nature in the cell environment and that they are safe to inject in a host. It reduces the probability of having allergic and negative responses by manufacturing the vaccine without damaging the host DNA material. Thus, this study focused on introducing the mRNA vaccine using E glycoprotein through computational methods. The reverse vaccinology approaches were used to identify the best possible T and B lymphocytes epitopes, where we obtained 1 CTL, 5 HTL, and 5 LBL promising epitopes. A peptide vaccine construct was designed using these predicted epitopes. This peptide construct was evaluated using different tools, which indicate the potential of the construct to elicit the human immune system. Our designed mRNA vaccine construct, i.e., ‘CHIKV-mRNAV’, is a prospective model prepared for further investigations, involving in vitro and then in vivo studies, to develop a successful CHIKV vaccine in the upcoming years.

## Figures and Tables

**Table 1:** Analysis of MHC-I binding T-cell epitopes.

Alleles	Start	End	Epitopes	Score	Rank	Conservancy	Antigenicity	Toxicity	Allergenicity
HLA-C*14:02	186	194	YYNWHHGAV	0.553042	0.15	100.00%	Antigen	Non-Toxin	Allergen
HLA-B*58:02	253	261	ITPEGAEEW	0.191746	0.06	88.89%	Non-Antigen	Non-Toxin	Allergen
HLA-C*15:02	1170	1178	ASAEFRVQV	0.910008	0.01	77.78%	Antigen	Non-Toxin	Non-Allergen
HLA-B*35:03	772	780	IPLAALIVL	0.819695	0.05	88.89%	Antigen	Non-Toxin	Allergen
HLA-C*07:01	719	727	RRCITPYEL	0.309353	0.05	88.89%	Antigen	Non-Toxin	Non-Allergen
HLA-A*24:02	195	203	QYSGGRFTI	0.779379	0.06	88.89%	Non-Antigen	Non-Toxin	Allergen
HLA-A*30:02	653	661	VTWGNNEPY	0.46361	0.2	77.78%	Antigen	Non-Toxin	Allergen
HLA-C*12:03	672	680	TAHGHPHEI	0.919117	0.01	77.78%	Non-Antigen	Non-Toxin	Non-Allergen
HLA-A*32:01	888	896	KVFTGVYPF	0.959162	0.01	88.89%	Non-Antigen	Non-Toxin	Allergen
HLA-B*35:01	816	824	IPNTVGVPY	0.986276	0.01	88.89%	Antigen	Non-Toxin	Allergen
HLA-C*15:02	1222	1230	ITGGVGLVV	0.442712	0.17	88.89%	Antigen	Non-Toxin	Allergen
HLA-B*51:01	430	438	CPKGETLTV	0.706451	0.07	88.89%	Antigen	Non-Toxin	Non-Allergen
HLA-B*08:01	219	227	DNKGRVVAI	0.789156	0.04	88.89%	Antigen	Non-Toxin	Allergen
HLA-C*14:02	683	691	YYYELYPTM	0.960772	0.01	88.89%	Antigen	Non-Toxin	Allergen
HLA-A*33:01	385	393	DSHDWTKLR	0.815075	0.03	88.89%	Non-Antigen	Non-Toxin	Allergen
HLA-C*15:02	1140	1148	HSMTNAVTI	0.500844	0.14	88.89%	Non-Antigen	Non-Toxin	Non-Allergen
HLA-C*14:02	612	620	LYPDHPTLL	0.969246	0.01	88.89%	Non-Antigen	Non-Toxin	Non-Allergen
HLA-B*08:01	1052	1060	WLKERGASL	0.974106	0.01	88.89%	Antigen	Non-Toxin	Non-Allergen
HLA-A*68:02	1062	1070	HTAPFGCQI	0.79801	0.05	88.89%	Antigen	Non-Toxin	Allergen
HLA-B*08:01	593	601	VPKARNPTV	0.792978	0.04	88.89%	Non-Antigen	Non-Toxin	Non-Allergen
HLA-C*15:02	845	853	VTLEPTLSL	0.887273	0.01	88.89%	Antigen	Non-Toxin	Non-Allergen
*HLA-C*04:01	**159**	**167**	**KYDLECAQI**	**0.366935**	**0.11**	**100.00%**	**Antigen**	**Non-Toxin**	**Non-Allergen**

* The row in bold represents the final selected peptide.

**Table 2:** MHC-II binding T-cell epitopes analysis.

Alleles	Start	End	Peptide	Rank	Conservancy	Antigenicity	Toxicity	Allergenicity	IL4 Inducer	IL10 Inducer
HLA-DPA1*01:03/DPB1*02:01	677	691	PHEIILYYYELYPTM	0.04	66.67%	Antigen	Non-Toxin	Non-Allergen	IL4 inducer	IL10 non-inducer
HLA-DQA1*05:01/DQB1*03:01	1219	1233	VQKITGGVGLVVAVA	0.85	86.67%	Non-Antigen	Non-Toxin	Non-Allergen	Non IL4 inducer	IL10 non-inducer
HLA-DRB1*09:01	723	737	TPYELTPGATVPFLL	0.88	93.33%	Antigen	Non-Toxin	Non-Allergen	Non IL4 inducer	IL10 non-inducer
*HLA-DRB1*13:02	**213**	**227**	**SGRPIFDNKGRVVAI**	**2**	**93.33%**	**Antigen**	**Non-Toxin**	**Non-Allergen**	**IL4 inducer**	**IL10 non-inducer**
HLA-DQA1*01:02/DQB1*06:02	230	244	GGANEGARTALSVVT	1.1	93.33%	Non-Antigen	Non-Toxin	Allergen	IL4 inducer	IL10 non-inducer
HLA-DRB3*02:02	405	419	RAGLFVRTSAPCTIT	1.1	86.67%	Antigen	Non-Toxin	Non-Allergen	IL4 inducer	IL10 non-inducer
HLA-DQA1*01:02/DQB1*06:02	1137	1151	CAVHSMTNAVTIREA	0.86	93.33%	Non-Antigen	Non-Toxin	Non-Allergen	Non IL4 inducer	IL10 non-inducer
HLA-DRB1*04:04	1234	1248	ALILIVVLCVSFSRH	1.1	93.33%	Antigen	Non-Toxin	Allergen	Non IL4 inducer	IL10 inducer
*HLA-DRB3*01:01	**103**	**117**	**ERMCMKIENDCIFEV**	**2**	**100.00%**	**Antigen**	**Non-Toxin**	**Non-Allergen**	**IL4 inducer**	**IL10 inducer**
HLA-DRB3*02:02	557	571	HKKWQYNSPLVPRNA	1.6	86.67%	Antigen	Non-Toxin	Non-Allergen	IL4 inducer	IL10 non-inducer
*HLA-DRB1*04:05	**856**	**870**	**ITCEYKTVIPSPYVK**	**1.4**	**93.33%**	**Antigen**	**Non-Toxin**	**Non-Allergen**	**IL4 inducer**	**IL10 non-inducer**
*HLA-DRB3*01:01	**988**	**1002**	**VYKGDVYNMDYPPFG**	**1.8**	**93.33%**	**Antigen**	**Non-Toxin**	**Non-Allergen**	**IL4 inducer**	**IL10 non-inducer**
HLA-DRB1*11:01	821	835	GVPYKTLVNRPGYSP	1.4	93.33%	Non-Antigen	Non-Toxin	Allergen	Non IL4 inducer	IL10 inducer
HLA-DPA1*02:01/DPB1*14:01	929	943	SAYRAHTASASAKLR	0.74	86.67%	Antigen	Non-Toxin	Non-Allergen	Non IL4 inducer	IL10 non-inducer
HLA-DRB3*02:02	599	613	PTVTYGKNQVIMLLY	1.7	86.67%	Antigen	Non-Toxin	Allergen	Non IL4 inducer	IL10 non-inducer
HLA-DRB4*01:01	365	379	EATDGTLKIQVSLQI	0.61	93.33%	Antigen	Non-Toxin	Non-Allergen	Non IL4 inducer	IL10 non-inducer
HLA-DRB5*01:01	1039	1053	HVPYSQAPSGFKYWL	0.37	93.33%	Non-Antigen	Non-Toxin	Non-Allergen	IL4 inducer	IL10 non-inducer
HLA-DRB1*07:01	1155	1169	VEGNSQLQISFSTAL	0.27	86.67%	Antigen	Non-Toxin	Non-Allergen	IL4 inducer	IL10 inducer
*HLA-DRB1*03:01	**126**	**140**	**YACLVGDKVMKPAHV**	**1.1**	**93.33%**	**Antigen**	**Non-Toxin**	**Non-Allergen**	**IL4 inducer**	**IL10 inducer**
HLA-DRB1*12:01	146	160	NADLAKLAFKRSSKY	1.9	86.67%	Antigen	Non-Toxin	Non-Allergen	IL4 inducer	IL10 non-inducer

* The rows in bold represent the final selected peptides.

**Table 3:** Predicted linear B-cell epitopes analysis.

Start	End	Epitopes	Score	Antigenicity	Allergenicity	Toxicity	Conservancy
198	217	GGRFTIPTGAGKPGDSGRPI	1	Non-Antigen	Non-Allergen	Non-Toxin	95.00%
993	1012	VYNMDYPPFGAGRPGQFGDI	1	Antigen	Allergen	Non-Toxin	90.00%
490	509	EEIEVHMPPDTPDRTLMSQQ	0.996	Non-Antigen	Non-Allergen	Non-Toxin	85.00%
447	466	SHSCTHPFHHDPPVIGREKF	0.983	Antigen	Non-Allergen	Toxin	80.00%
242	261	VVTWNKDIVTKITPEGAEEW	0.98	Non-Antigen	Allergen	Non-Toxin	90.00%
**1058**	**1077**	*ASLQHTAPFGCQIATNPVRA	**0.976**	**Antigen**	**Non-Allergen**	**Non-Toxin**	**95.00%**
**972**	**991**	*VGPMSSAWTPFDNKIVVYKG	**0.974**	**Antigen**	**Non-Allergen**	**Non-Toxin**	**95.00%**
**177**	**196**	*KFTHEKPEGYYNWHHGAVQY	**0.968**	**Antigen**	**Non-Allergen**	**Non-Toxin**	**95.00%**
654	673	TWGNNEPYKYWPQLSTNGTA	0.958	Non-Antigen	Non-Allergen	Non-Toxin	90.00%
1149	1168	REAEIEVEGNSQLQISFSTA	0.925	Antigen	Non-Allergen	Non-Toxin	85.00%
856	875	ITCEYKTVIPSPYVKCCGTA	0.917	Non-Antigen	Allergen	Toxin	80.00%
807	826	VSAYEHVTVIPNTVGVPYKT	0.912	Antigen	Non-Allergen	Non-Toxin	90.00%
1037	1056	TVHVPYSQAPSGFKYWLKER	0.875	Non-Antigen	Non-Allergen	Non-Toxin	95.00%
883	902	PDYSCKVFTGVYPFMWGGAY	0.819	Antigen	Allergen	Non-Toxin	95.00%
**360**	**379**	*ERIRNEATDGTLKIQVSLQI	**0.812**	**Antigen**	**Non-Allergen**	**Non-Toxin**	**95.00%**
**715**	**734**	*MCARRRCITPYELTPGATVP	**0.757**	**Antigen**	**Non-Allergen**	**Non-Toxin**	**95.00%**
129	148	LVGDKVMKPAHVKGTIDNAD	0.752	Non-Antigen	Allergen	Non-Toxin	95.00%

* The rows in bold represent the final selected peptides.

**Table 4:** Analysis of peptide vaccine construct.

Physiochemical Properties	Measurement	Indication
Total Number of Amino Acids	631	Appropriate
Molecular Weight	70,036.77	Appropriate
Theoretical pI	8.52	Basic
Total Number of Negatively Charged Residues (Asp + Glu)	82	-
Total Number of Positively Charged Residues (Arg + Lys)	90	-
Aliphatic Index (AI)	80.55	Thermostable
Grand Average of Hydropathicity (GRAVY)	–0.361	Hydrophilic
Antigenicity (using Vaxijen)	0.4711	Antigenic
Solubility upon overexpression	0.722294	Soluble
Allergenicity	Non-allergen	Non-Allergenic

## Data Availability

Not applicable.

## Supplementary Materials

The following supporting information can be downloaded at: https://www.mdpi.com/article/10.3390/vaccines10091476/s1, Figure S1: Prediction of possible secondary structures in peptide vaccine construct design by SOPMA server; Figure S2: Three-dimensional structural indication of highly scoring conformational B-cell epitopes using Ellipro server; Figure S3: Designed potential mRNA vaccine construct against CHIKV infection; Figure S4: In silico restriction cloning of final mRNA vaccine construct into the *E. coli* pET28a (+) expression vector, where red color shows the cloned vaccine construct sequence. Table S1: Non-overlapping MHC-I binding T-cell epitopes with rank ≤ 0.2; Table S2: Non-overlapping MHC-II binding T-cell epitopes with rank ≤ 2.0; Table S3: Population Coverage analysis by IEDB resource.; Table S4: Conformational B-cell epitopes prediction by Ellipro server.; Table S5: Analysis of mRNA sequence by codon optimization tools.
