# Supplementary material for: mRNA Vaccine Designing Using Chikungunya Virus E Glycoprotein through Immunoinformatics-Guided Approaches
Source: Vaccines (Basel). 2022 Sep 6;10(9):1476. doi: 10.3390/vaccines10091476 (PMC9500984; doi:10.3390/vaccines10091476)
Supplement: Supplementary file 1 [file vaccines-10-01476-s001.zip › Supplementary Table S3.pdf]

Supplementary Table S3. Population Coverage analysis by IEDB resource.

Sr. No.	T-cell epitopes	Interacting MHC alleles	Highly interacting MHC allele list	Population Coverage (South Asia)
1	KYDLECAQI	MHC-I	HLA-C*04:01, HLA-A*24:02, HLA-A*23:01, HLA-C*14:02, HLA-C*08:02	39.06%
2	SGRPIFDNKGRVVAI	MHC-II	HLA-DRB1*13:02	3.59%
3	ERMCMKIENDCIFEV	MHC-II	HLA-DRB1*03:01	16.37%
4	ITCEYKTVIPSPYVK	MHC-II	HLA-DRB1*04:05, HLA-DRB1*09:01	3.20%
5	VYKGDVYNMDYPPFG	MHC-II	HLA-DRB1*04:01	2.76%
6	YACLVGDKVMKPAHV	MHC-II	HLA-DRB1*03:01, HLA-DRB1*09:01	18.38%
