# Supplementary material for: mRNA Vaccine Designing Using Chikungunya Virus E Glycoprotein through Immunoinformatics-Guided Approaches
Source: Vaccines (Basel). 2022 Sep 6;10(9):1476. doi: 10.3390/vaccines10091476 (PMC9500984; doi:10.3390/vaccines10091476)
Supplement: Supplementary file 1 [file vaccines-10-01476-s001.zip › Supplementary Table S4.pdf]

Supplementary Table S4. Conformational B-cell epitopes prediction by Ellipro server.

Sr. No.	Residues	No. of residues	Score
1	A:I355, A:Y358, A:E359, A:L360, A:T361, A:P362, A:G363, A:A364, A:T365, A:V366, A:P367, A:A368, A:A369, A:Y370, A:K371, A:Y372, A:D373, A:L374, A:E375, A:C376, A:Q378	21	0.821
2	A:I211, A:P212, A:S213, A:P214, A:Y215, A:V216, A:K217, A:G218, A:P219, A:G220, A:P221, A:G222, A:Y224, A:K225, A:G226, A:D227, A:V228, A:Y229, A:N230, A:M231, A:D232, A:Y233, A:P234, A:P235, A:F236, A:G237, A:G238, A:P239, A:G240, A:P241, A:G242, A:Y243, A:A244, A:C245, A:L246, A:V247, A:G248, A:D249, A:K250, A:V251, A:M252, A:K253, A:P254, A:A255, A:H256, A:V257, A:K258, A:K259, A:A260, A:S261, A:L262, A:Q263, A:H264, A:K281, A:V282, A:G283, A:P284, A:M285, A:S286, A:S287, A:A288	61	0.787
3	A:G269, A:C270, A:Q271, A:I272, A:A273, A:T274, A:N275, A:P276, A:V277, A:R278, A:A279, A:K280	12	0.782
4	A:G7, A:L8, A:C9, A:C10, A:V11, A:L12, A:L13, A:L14, A:C15, A:G16, A:A17, A:V18, A:F19, A:M43, A:T44, A:L46, A:E47, A:L48, A:S49, A:D50, A:F51, A:V52, A:K53, A:K54, A:F55, A:E56, A:E57, A:T58, A:F59, A:V61, A:T62, A:A63, A:A64, A:A65, A:P66, A:V67, A:A68, A:V69, A:A70, A:A71, A:A72, A:G73, A:A74	43	0.727
5	A:Q87, A:S88, A:F90, A:D91, A:V92, A:I93, A:L94, A:E95, A:A96, A:A97, A:G98, A:D99, A:K100, A:K101, A:I102, A:G103, A:V104, A:I105, A:K106, A:L147, A:E148, A:A149, A:A150, A:G151, A:A152, A:T153, A:V154, A:T155, A:V156, A:K157, A:G158	31	0.72
6	A:M1, A:D2, A:A3, A:M4, A:K5, A:R6, A:V20, A:S21, A:P22, A:E23, A:A24, A:A25, A:A26, A:K27, A:M28, A:A29, A:K30, A:L31, A:S32	19	0.712
7	A:D467, A:G468, A:K469, A:H471, A:N479, A:A480, A:T481, A:G482, A:F483, A:S484, A:E486, A:S487, A:R490, A:E491, A:Y492, A:L493, A:N494, A:E495, A:T496, A:V497, A:T498, A:E499, A:V500, A:W501, A:P505, A:I507, A:R508, A:H509, A:T510, A:L513, A:Y514, A:F516, A:L517, A:R518, A:C520, A:E521, A:M522, A:L523, A:I524, A:K525, A:R526, A:P527, A:C528, A:K529, A:V530, A:K531, A:T532, A:I533, A:H534, A:L535, A:L536, A:T537, A:S538, A:L539, A:D540, A:E541, A:G542, A:I543, A:E544, A:Q545, A:V546, A:Q547, A:Q548, A:S549, A:R550, A:G551, A:L552, A:Q553, A:E554, A:I555, A:E556, A:E557, A:S558, A:L559, A:R560, A:S561, A:H562, A:G563, A:V564, A:L565, A:L566, A:E567, A:V568, A:Q569, A:Y570, A:S571, A:S572, A:S573, A:I574, A:R580, A:F581, A:N582, A:N583, A:G584, A:W585, A:F596, A:K597, A:K598, A:P599, A:Q600, A:S601, A:R602, A:F603, A:S604, A:L605, A:G606, A:Y607, A:C608, A:D609, A:F610, A:D611, A:L612, A:R613, A:P614, A:C615, A:H616, A:H624, A:K625, A:K626, A:H627, A:T628, A:K629, A:N630, A:I631	124	0.686
8	A:D462, A:Q463, A:K465	3	0.568
9	A:P161, A:G162, A:G178, A:P179, A:G180, A:P181, A:G182, A:E183, A:R184, A:M185, A:V197, A:G198, A:P199, A:G200, A:P201, A:G202, A:I203	17	0.543
