# Supplementary material for: mRNA Vaccine Designing Using Chikungunya Virus E Glycoprotein through Immunoinformatics-Guided Approaches
Source: Vaccines (Basel). 2022 Sep 6;10(9):1476. doi: 10.3390/vaccines10091476 (PMC9500984; doi:10.3390/vaccines10091476)
Supplement: Supplementary file 1 [file vaccines-10-01476-s001.zip › Supplementary Table S5.pdf]

Supplementary Table S5. Analysis of mRNA sequence by codon optimization tools

Optimization tool	Average GC content (%)	CAI value
JCAT tool	66.51	0.96
ExpOptimizer tool	50.13%	0.81
