# Supplementary figures and images for: mRNA Vaccine Designing Using Chikungunya Virus E Glycoprotein through Immunoinformatics-Guided Approaches
Source: Vaccines (Basel). 2022 Sep 6;10(9):1476. doi: 10.3390/vaccines10091476 (PMC9500984; doi:10.3390/vaccines10091476)

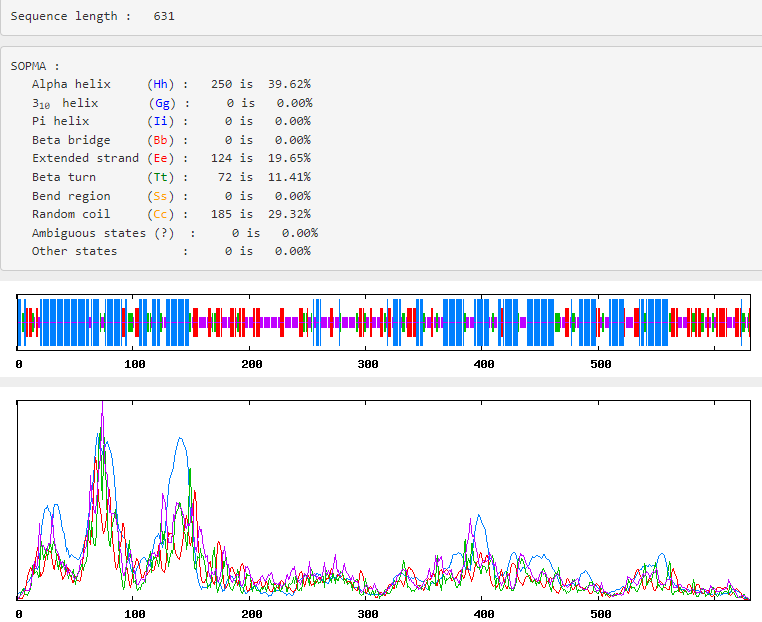

Supplement: Supplementary file 1 [file vaccines-10-01476-s001.zip › Supplementary Figure S1.PNG]

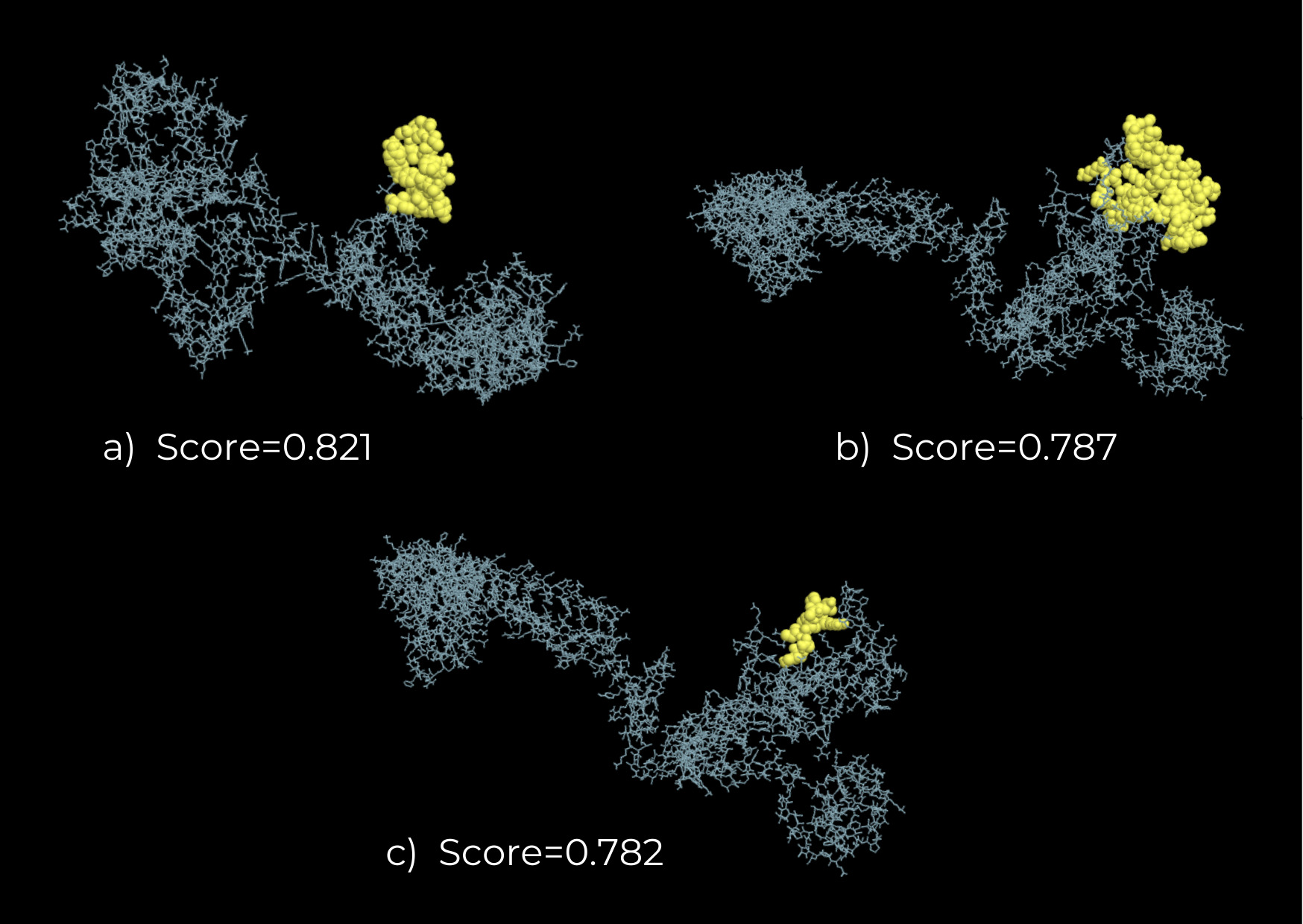

Supplement: Supplementary file 1 [file vaccines-10-01476-s001.zip › Supplementary Figure S2.jpg]

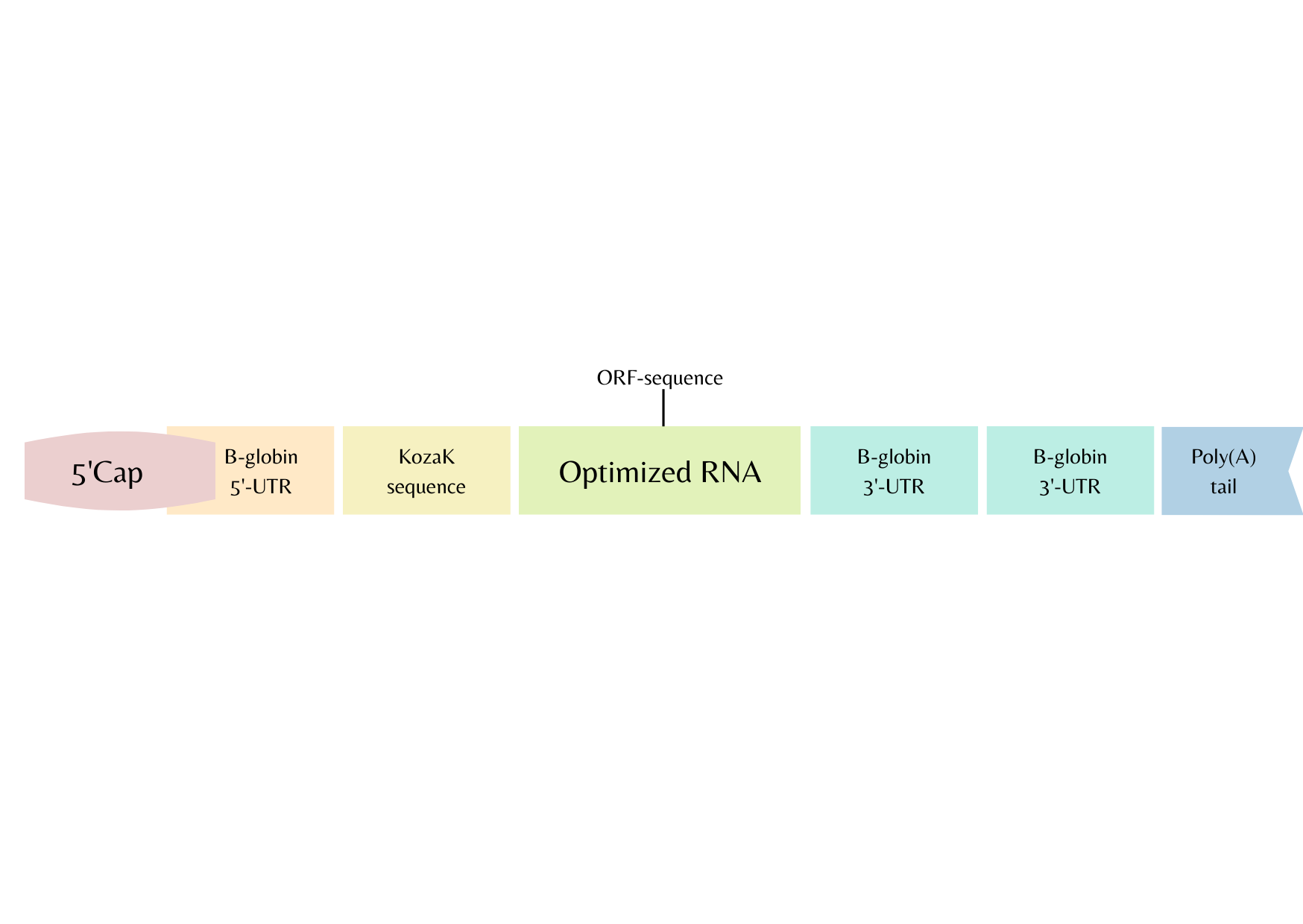

Supplement: Supplementary file 1 [file vaccines-10-01476-s001.zip › Supplementary Figure S3.png]

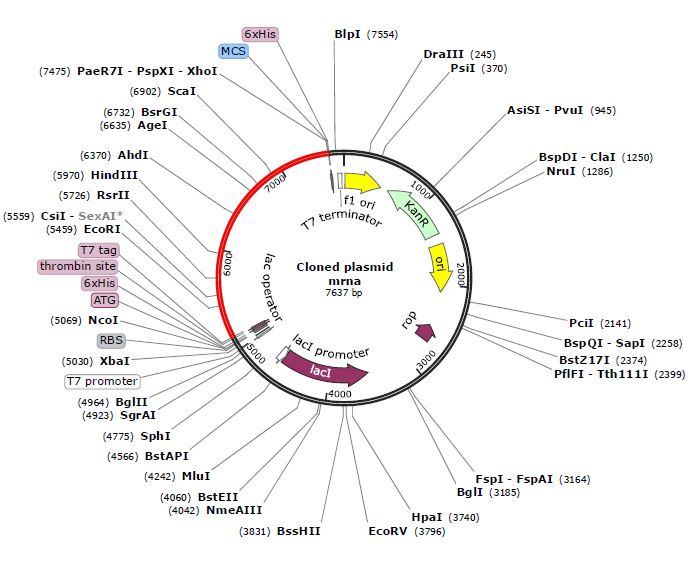

Supplement: Supplementary file 1 [file vaccines-10-01476-s001.zip › Supplementary Figure S4.JPG]
